# Early effect of Botox-A injection into the masseter muscle of rats: functional and histological evaluation

**DOI:** 10.1186/s40902-015-0049-6

**Published:** 2015-12-29

**Authors:** Young-Min Moon, Young-Jun Kim, Min-Keun Kim, Seong-Gon Kim, HaeYong Kweon, Tae-Woo Kim

**Affiliations:** 1 Department of Orthodontics, School of Dentistry, Dental Research Institute, Seoul National University, Seoul, South Korea; 2 Department of Oral Medicine and Diagnosis, College of Dentistry, Research Institute of Oral Science, Gangneung-Wonju National University, Gangneung, South Korea; 3 Department of Oral and Maxillofacial Surgery, College of Dentistry, Gangneung-Wonju National University, 7 Jukhyun-gil, Gangneung, 210-702 South Korea; 4 Sericultural and Apicultural Materials Division, National Academy of Agricultural Science, Suwon, South Korea

**Keywords:** Botulinum toxin A, Masseter muscle, Myosin type II, Food intake

## Abstract

**Background:**

The purpose of this study was to evaluate the change of food intake after different dosages of botulinum toxin A (BTX) injection in the animal model. Additionally, the dimensional and histological change at 14 days after BTX injection was also evaluated.

**Methods:**

The comparative study was performed using the BTX injection model in rats (*n* = 5 for each group). Group 1 was the saline-injected group. Group 2 was the 5-unit BTX-injection group to each masseter muscle. Group 3 was the 10-unit BTX-injection group to each masseter muscle. Food intake rates and body weight were checked daily before and after BTX injection until 10 days. All animals were sacrificed at 14 days after BTX injection, and the specimens underwent hematoxylin and eosin stain and immunohistochemical staining for myosin type II (MYH2).

**Results:**

The recovery of food intake in groups 2 and 3 decreased significantly compared with group 1 from day 2 to day 7 and day 9 after injection (*p* < 0.05). The BTX-treated masseter muscles were significantly smaller than those in group 1 (*p* = 0.015). The immunohistochemical findings demonstrated that the expression of MYH2 was significantly higher in group 3 compared to groups 1 and 2 (*p* < 0.001).

**Conclusions:**

BTX injection to the masseter muscle in rats demonstrated short food-intake-rate reduction with recovery until 10 days after injection. The thickness of the masseter muscle and MYH2 expression were significantly changed according to the injected dose of BTX.

## Background

Botulinum toxin A (BTX), a toxin produced by *Clostridium botulinum*, binds to snail protein in the presynaptic cholinergic nerve and inhibits the acetylcholine release [1]. As the neurotransmitter of the motor nerve is acetylcholine, BTX can be used for intentional skeletal muscle weakening [2]. BTX can be used to decrease the hyperactivity of the masseter and the temporal muscles for reducing painful conditions [2]. Myofascial pain is described as a muscle hyperactivity involving facial pain related to temporomandibular disorders [3]. Tension headaches and neck pain are usually caused by masticatory muscle hyperactivity [4]. Secretion of the salivary gland and sweat gland is also controlled by the cholinergic nerve [5]. Accordingly, BTX has been widely used in the dental field. BTX is used for the treatment of temporomandibular disorders [2], sialorrhea [5], post-traumatic open bite [6], and masseteric muscle hypertrophy [7, 8].

To achieve optimal results, optimal dosage of BTX injection should be important. For the treatment of the masseter muscle hypertrophy, 25 to 30 U of BTX has been generally given for each side [7, 9]. The maximum bite force is reduced after injection of BTX into the masseter muscle [10]. Complications of BTX injection into the masseter muscle, such as a temporary change in the bite force, have been reported [7, 8].

There have been several reports to investigate the effect of BTX injection into the masseter muscle in the animal model [11–14]. The injection of BTX immediately reduces the masseter muscle activity measured by electromyogram [12]. BTX changes the component of muscle fiber and morphology of the mandible at 1 month after injection [13, 14]. However, early changes of muscle fiber component have not been studied. Post-traumatic open bite is corrected within a couple of days after BTX injection [6]. Some patients showed temporary muscle weakness immediately after BTX injection [7, 8]. Thus, early change after BTX injection on the masseter muscle is important to understand the clinical application of BTX.

The purpose of this study was to evaluate the change of food intake after different dosages of BTX injection in the animal model. Additionally, dimensional and histological change at 14 days after BTX injection was also evaluated.

## Methods

### Animals and experimental design

Male Wistar rats aged 18 weeks were purchased from Samtako (Seoul, Korea). They were housed individually in controlled temperature (20–22 °C) and hygrometry (around 40 %) in a 12-h light:12-h darkness cycle. They had free access to water. During the adaptation period (first week), all rats were fed ad libitum with a control semi-synthetic diet (4 % lipids from soya vegetal oil, 74 % carbohydrates from sucrose and cornstarch, and 14 % proteins from casein, supplemented with standard vitamins and mineral mix), following classical recommendations. All diets were prepared within Gangneung-Wonju National University facilities. All groups were maintained ad libitum for 7 days receiving a diet similar to the adaptation diet with measuring of daily spontaneous intake (26.1 ± 4.1 g/day, *n* = 15). At the end of the normal diet period, rats (20 weeks old) were separated: the control group received a saline injection into both masseter muscles (group 1, *n* = 5), and the others were separated in two groups for BTX injection study (*n* = 5 per group). These two groups were assessed in order to compare the dose-dependent effect of BTX injection on physiological parameters in two animal groups receiving re-feeding diets. All re-feeding diets were the same to the ad libitum control period. In order to measure food intake, all groups were individually housed. Group 1 was the saline-injected group. Group 2 was the 5-unit BTX-injection group to each masseter muscle. Group 3 was the 10-unit BTX-injection group to each masseter muscle. The recovery of food intake was measured until 10 days after the injection. All animals were sacrificed at 14 days after the injection for the histological analysis. All procedures were conducted according to the guidelines of laboratory animal care and were approved by the Gangneung-Wonju National University for animal research (GWNU-2015-24).

### Histomorphometric evaluation

The samples were harvested, decalcified in 5 % nitric acid for 5 days, and dehydrated in ethyl alcohol and xylene. After separation of the calvarial bones, the samples were embedded in paraffin blocks. The paraffin blocks were sliced into sections that were then stained with hematoxylin and eosin. The section with the occlusal plane area was selected.

The staining procedure for hematoxylin and eosin staining was as follows. First, de-wax and hydrate paraffin sections. The slide was stained in hematoxylin for 5 min. Overstained sections can easily be differentiated by agitating for a second in acid-alcohol then washing in tap water for 5 min. The slides were immersed in eosin for 30 s and then washed in running tap water for 1 min. The slides were dehydrated and cleared in xylene.

Digital images of the selected sections were captured with a digital camera (DP-73; Olympus, Tokyo, Japan). The images were analyzed by Sigma Scan pro (SPSS, Chicago, IL). The thickness of the masseter muscle was measured from the perpendicular line to the mandibular ramus.

### Immunohistochemical determination of lysozyme and myosin type II in rat masseter muscle

To determine the level of expression of myosin type II (MYH2), immunohistochemical staining was performed using anti-myosin type II antibodies (Santa Cruz Biotech, Santa Cruz, CA, USA). Paraffin-embedded tissues from rat masseter muscles were prepared. For antigen retrieval, sections were incubated in trypsin for 7 min at 37 °C. The primary antibody dilutions were as follows: MYH2, 1:50. The immunohistochemical procedures were performed as described in a previous publication [15]. The negative controls were sections stained without primary antibodies.

Stained sections were examined in an Olympus BX51 (Olympus, Tokyo, Japan) microscope. To quantify the immunohistochemical reaction intensity, the positive intensity MYH2 staining in 10 random fields at ×200 magnification in the masseter muscle was evaluated by computer-assisted image analysis after image transformation to grayscale. The staining intensity was expressed as the mean intensity value (0, no stain; 255, highest stain). The samples were not counterstained so that the absorbance would be solely attributable to the product of the immunohistochemical reaction.

### Statistical analysis

SPSS for Windows ver. 19 (IBM Co., Armonk, NY, USA) were used for statistical analysis. The differences among groups were evaluated by ANOVA. For post hoc tests, Bonferroni’s method was used. The statistically significant level was set as *p* < 0.05.

## Results

**Fig. 1:**
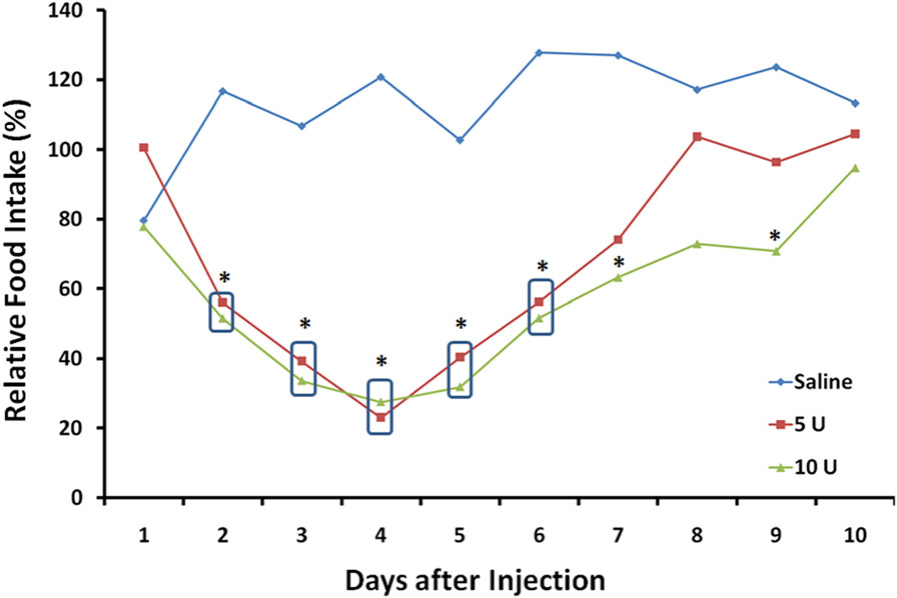
The relative amount of food consumption during 24 h compared to before the injection. The recovery of food intake in rats treated with BTX (5–10 U) decreased significantly compared with the saline group from day 2 to day 7 and day 9 after injection (*asterisk*, *p* < 0.05)

There was no initial difference between the BTX and saline solution groups in any parameter, including body weight and the amount of food consumption during 24 h. Figure 1 illustrates the relative amount of food consumption during the 24 h compared to before the injection. The relative amount of food intake in rats treated with BTX (5–10 U) decreased significantly compared with the saline group from day 2 to day 7 and day 9 after injection (*p* < 0.05). In the post hoc test, both 5- and 10-U BTX treatments resulted in significantly lower values compared with the saline-treated control group from day 2 to day 6 (*p* < 0.05). However, only the 10-U BTX treatment resulted in significantly lower values compared with the saline-treated control group at day 7 and day 9 (*p* = 0.031 and 0.049, respectively). There was no significant difference in body weight between the observation points (data not shown).

**Fig. 2:**
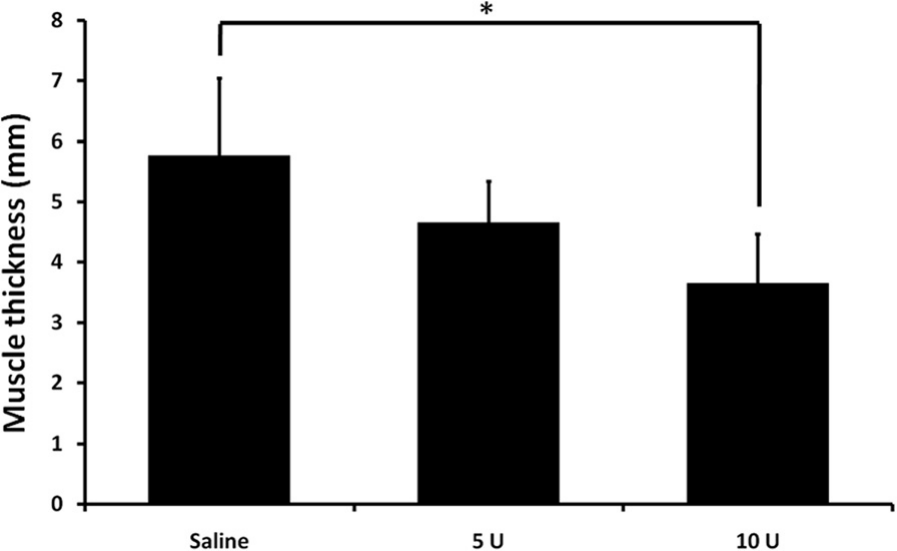
The thickness of the masseter muscle. The thickness of the masseter muscle in rats treated with BTX (5–10 U) was decreased significantly compared with the saline group at day 14 after injection (*asterisk*, *p* = 0.015)

**Fig. 3:**
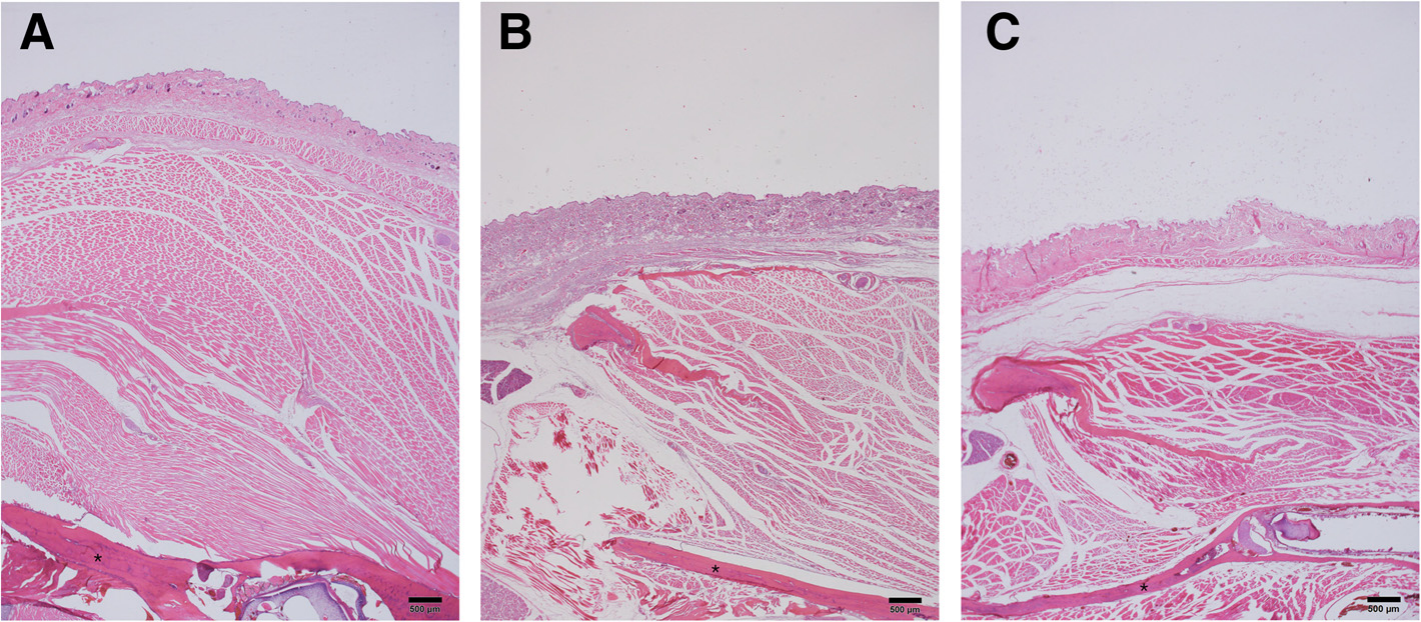
Histological view. **a** The saline-treated group, **b** 5-U BTX-treated group, **c** 10-U BTX-treated group. Interestingly, the thickness of the mandibular ramus (*asterisk*) was changed after BTX injection. Degenerative change was also shown in both the 5- and 10-U BTX-treated groups (hematoxylin and eosin stain, original magnification ×20)

The BTX-treated masseter muscles were significantly smaller than the saline-solution-injected masseter muscles, as shown in Figs. 2 and 3. The thickness of the masseter muscle was 5.76 ± 1.28 mm, 4.66 ± 0.68 mm, and 3.65 ± 0.81 mm for the saline, 5-U, and 10-U BTX treatments, respectively (Fig. 2; *p* = 0.015). The post hoc test revealed differences between the group treated with 10 U BTX and the group treated with saline (control) resulting in significantly lower values (*p* = 0.013). In the histological view, the degenerative change of muscle fibers was prominent in the 10-U BTX-treated group. Inter-fiber spaces were lost in the 10-U BTX-treated group. Mild degenerative change was also noticed in the 5-U BTX-treated group (Fig. 3).

**Fig. 4:**
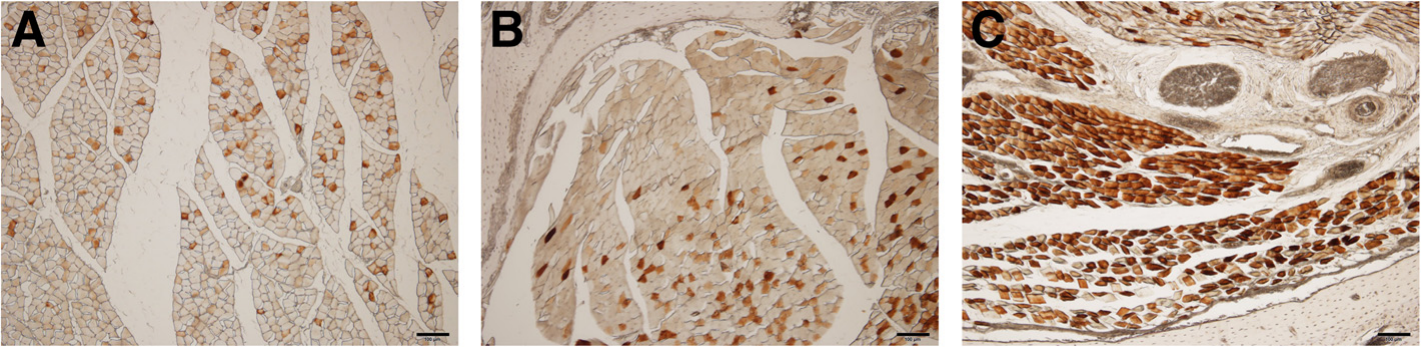
Immunohistological view. **a** The saline-treated group, **b** 5-U BTX-treated group, **c** 10-U BTX-treated group. The immunohistochemical findings demonstrated that the expression of MYH2 was much higher in the 10-U BTX-treated group compared to the saline group (original magnification ×100 without counterstain)

The immunohistochemical findings demonstrated that the expression of MYH2 was much higher in the 10-U BTX-treated group compared to the saline group (Fig. 4). The mean intensity of MYH2 was 86.38 ± 8.66, 101.72 ± 12.41, and 144.51 ± 5.68 for the saline, 5-U, and 10-U BTX treatments, respectively (Fig. 4; *p* < 0.001). The post hoc test revealed differences between the groups treated with 10 U BTX, resulting in significantly higher values compared with the saline-treated control and the 5-U BTX-treated group (*p* < 0.001).

## Discussion

### Recovery after BTX injection

In this study, the recovery in food intake after BTX injection was taken approximately 10 days after injection (Fig. 1). When BTX is injected into the unilateral masseter muscle of an adult rabbit, the relative bite force is reduced over 85 % in 3 weeks after injection [16]. Thus, 10 days might be a relatively fast recovery. After BTX injection of the unilateral masseter muscle, rabbits still masticate at the same rates and chew on both sides [16]. At 12 weeks after unilateral injection, bite force has returned to pre-injection levels but the BTX-injected masseter muscle volume is still smaller than the contralateral muscle [16]. This might have been due to the compensatory hyperfunction of the temporalis muscle to adjust to the masticatory function [17]. In this study, the other masticatory muscles were intact except for the masseter muscle. Thus, early recovery of food intake rate might be due to the compensatory function of the other masticatory muscles.

The clinical literature indicates that maximal atrophy of the masseter of human is observed about 2 months after BTX injection and is sometimes persistent until 1 to 2 years [18, 19]. However, voluntary bite force is subjectively normal within 8 days after BTX injection in one report [20]. Patients report only brief periods of problems with chewing after BTX injection and the return of normal function after a short time [21]. Our results demonstrated similar results with previous publications (Fig. 1).

### Muscle and bone changes after BTX injection

The dosage of BTX expected to cause muscle paralysis is 20–30 U for rodents [22]; therefore, 5 and 10 U were used in this experiment. Injections of BTX decrease the force of masseter muscle contractions and decrease tension on the periosteum [23]. The BTX-treated masseter muscles were significantly smaller than the saline-solution-injected masseter muscles, as shown in Fig. 2. The masseter muscle may be involved in maintaining mandibular bone volume through changes in bone metabolism [22]. Accordingly, bony deposition decreases and a morphological change are induced [24]. A reduced cortical bone thickness of the mandibular ramus in our study (Fig. 3) was also consistent with the research of previous publications [25, 26]. The diminished forces cause the remodeling process of bone growth, which circumferentially reduces the cortical bone thickness. When BTX is used on the limb muscle, bone loss is observed in the tibia and distal femur [27, 28].

### MYH change after BTX injection

MYH is found in all eukaryotic cells, where it provides the motor function [29]. There are several types of MYH, and they can be classified by their contraction speed as fast type and slow type [29]. MYH2 is fast type [30]. Masticatory muscle fibers can adapt to stress by changing their myosin composition, and they are found after orthognathic surgery of patients with malocclusions [31, 32]. The patients having mandibular asymmetry are associated with a significant increase of MYH2 [33]. In addition, MYH2 occupancy is increased in deep bites [34].

It was shown that intramuscular BTX application induces direct and significant alterations of the fiber composition [35]. Just like BTX injection, chronic denervation is followed by changes of the fiber type composition and MYH content in rat muscles [36, 37]. Endurance muscle training induced by sagittal advancement of the mandible induces an increase of MYH1 in pigs [38]. MYH2 is increased in the masseter muscle after BTX injection [39]. In our study, MYH2 was significantly increased in the masseter muscle after BTX injection and its increasing was BTX dose dependent (Fig. 4). Unlike BTX injection, sleep deprivation is associated with significantly decreased MYH2 in the masseter muscle in rats [40]. Though our study indicated that BTX injection could increase MYH2 occupancy in the masseter muscle, because human masseter and temporal muscles have also heterogeneous fiber-type composition, the proportion of MYH2 being generally smaller [41], there will be needed translational research in humans.

## Conclusions

In conclusion, BTX injection to the masseter muscle in rats demonstrated a short food-intake-rate reduction with recovery until 10 days after injection. The thickness of the masseter muscle and MYH2 expression were significantly changed according to the injected dose of BTX.

## Abbreviations

BTX: botulinum toxin A
MYH2: myosin type II

## References

[1] Kiris E, Burnett JC, Kane CD, Bavari S (2014). Recent advances in botulinum neurotoxin inhibitor development. Curr Top Med Chem.

[2] Tan EK, Jankovic J (2000). Treating severe bruxism with botulinum toxin. J Am Dent Assoc.

[3] Watts MW, Tan EK, Jankovic J (1999). Bruxism and cranial-cervical dystonia: is there a relationship?. Cranio.

[4] Molina OF, Dos Santos JJ, Nelson SJ, Grossman E (1997). Prevalence of modalities of headaches and bruxism among patients with craniomandibular disorder. Cranio.

[5] Santamato A, Ianieri G, Ranieri M, Megna M, Panza F, Fiore P (2008). Botulinum toxin type A in the treatment of sialorrhea in Parkinson’s disease. J Am Geriatr Soc.

[6] Seok H, Park YT, KimSG PYW (2013). Correction of post-traumatic anterior open bite by injection of botulinum toxin type A into the anterior belly of the digastric muscle: case report. J Korean Assoc Oral Maxillofac Surg.

[7] Kim HJ, Yum KW, Lee SS, Heo MS, Seo K (2003). Effects of botulinum toxin type A on bilateral masseteric hypertrophy evaluated with computed tomographic measurement. Dermatol Surg.

[8] Kim JH, Shin JH, Kim ST, Kim CY (2007). Effects of two different units of botulinum toxin type A evaluated by computed tomography and electromyographic measurements of human masseter muscle. Plast Reconstr Surg.

[9] von Lindern JJ, Niederhagen B, Appel T, Bergé S, Reich RH (2001). Type A botulinum toxin for the treatment of hypertrophy of the masseter and temporal muscles: an alternative treatment. Plast Reconstr Surg.

[10] Ahn KY, Kim ST (2007). The change of maximum bite force after botulinum toxin type A injection for treating masseteric hypertrophy. Plast Reconstr Surg.

[11] Kolaski K, Ajizian SJ, Passmore L, Pasutharnchat N, Koman LA, Smith BP (2008). Safety profile of multilevel chemical denervation procedures using phenol or botulinum toxin or both in a pediatric population. Am J Phys Med Rehabil.

[12] Park SY, Park YW, Ji YJ, Park SW, Kim SG (2015). Effects of a botulinum toxin type A injection on the masseter muscle: an animal model study. Maxillofac Plast Reconstr Surg.

[13] Tsai CY, Chiu WC, Liao YH, Tsaic CM (2009). Effects on craniofacial growth and development of unilateral botulinum neurotoxin injection into the masseter muscle. Am J Orthod Dentofacial Orthop.

[14] Kiliaridis S, Engstrom C, Thilander B (1988). Histochemical analysis of masticatory muscle in the growing rat after prolonged alteration in the consistency of the diet. Arch Oral Biol.

[15] Kim HC, Song JM, Kim CJ, Yoon SY, Kim IR, Park BS, Shin SH (2015). Combined effect of bisphosphonate and recombinant human bone morphogenetic protein 2 on bone healing of rat calvarial defects. Maxillofac Plast Reconstr Surg.

[16] Rafferty KL, Liu ZJ, Ye W, Navarrete AL, Nguyen TT, Salamati A, Herring SW (2012). Botulinum toxin in masticatory muscles: short- and long-term effects on muscle, bone, and craniofacial function in adult rabbits. Bone.

[17] Tsai CY, Lin YC, Su B, Yang LY, Chiu WC (2012). Masseter muscle fibre changes following reduction of masticatory function. Int J Oral Maxillofac Surg.

[18] Lee CJ, Kim SG, Kim YJ, Han JY, Choi SH, Lee SI (2007). Electrophysiologic change and facial contour following botulinum toxin A injection in square faces. Plast Reconstr Surg.

[19] Kim NH, Chung JH, Park RH, Park JB (2005). The use of botulinum toxin type A in aesthetic mandibular contouring. Plast Reconstr Surg.

[20] Freund B, Schwartz M, Symington JM (1999). The use of botulinum toxin for the treatment of temporomandibular disorders: preliminary findings. J Oral Maxillofac Surg.

[21] Yu CC, Chen PKT, Chen YR (2007). Botulinum toxin A for lower facial contouring: a prospective study. Aesthetic Plast Surg.

[22] Matic DB, Yazdani A, Wells RG, LeeTY GBS (2007). The effects of masseter muscle paralysis on facial bone growth. J Surg Res.

[23] Tsai CY, Yang LY, Chen KT, Chiu WC (2010). The influence of masticatory hypofunction on developing rat craniofacial structure. Int J Oral Maxillofac Surg.

[24] Kiliaridis S, Mejersjo C, Thilander B (1989). Muscle function and craniofacial morphology: a clinical study in patients with myotonic dystrophy. Eur J Orthod.

[25] Tsai CY, Huang RY, Lee CM, Hsiao WT, Yang LY (2010). Morphologic and bony structural changes in the mandible after a unilateral injection of botulinum neurotoxin in adult rats. J Oral Maxillofac Surg.

[26] Picard S, Lapointe NP, Brown JP, Guertin PA (2008). Histomorphometric and densitometric changes in the femora of spinal cord transected mice. Anat Rec (Hoboken).

[27] Poliachik SL, Bain SD, Threet D, Huber P, Gross TS (2010). Transient muscle paralysis disrupts bone homeostasis by rapid degradation of bone morphology. Bone.

[28] Warner SE, Sanford DA, Becker BA, Bain SD, Srinivasan S, Gross T (2006). Botox induced muscle paralysis rapidly degrades bone. Bone.

[29] Weiss A, Leinwand LA (1996). The mammalian myosin heavy chain gene family. Annu Rev Cell Dev Biol.

[30] Schiaffino S, Reggiani C (1996). Molecular diversity of myofibrillar proteins: gene regulation and functional significance. Physiol Rev.

[31] Gedrange T, Buttner C, Schneider M, Oppitz R, Harzer W (2005). Myosin heavy chain protein and gene expression in the masseter muscle of adult patients with distal or mesial malocclusion. J Appl Genet.

[32] Harzer W, Worm M, Gedrange T, Schneider M, Wolf P (2007). Myosin heavy chain mRNA isoforms in masseter muscle before and after orthognathic surgery. Oral Surg Oral Med Oral Pathol Oral Radiol Endod.

[33] Raoul G, Rowlerson A, Sciote J, Codaccioni E, Stevens L, Maurage CA, Duhamel A, Ferri J (2011). Masseter myosin heavy chain composition varies with mandibular asymmetry. J Craniofac Surg.

[34] Rowlerson A, Raoul G, Daniel Y, Close J, Maurage CA, Ferri J, Sciote J (2005). Fiber-type differences in masseter muscle associated with different facial morphologies. Am J Orthod Dentofacial Orthop.

[35] Borodic GE, Ferrante R, Pearce LB, Smith K (1994). Histologic assessment of dose-related diffusion and muscle fiber response after therapeutic botulinum A toxin injections. MovDisord.

[36] Bobinac D, Malnar-Dragojevic D, Bajek S, Soic-Vranic T, Jerkovic R (2000). Muscle fiber type composition and morphometric properties of denervated rat extensor digitorum longus muscle. Croat Med J.

[37] Del Gaudio JM, Sciote JJ (1997). Changes in myosin expression in denervated laryngeal muscle. Ann Otol Rhinol Laryngol.

[38] Gedrange T, Luck O, Hesske G, Buttner C, Seibel P, Harzer W (2001). Differential expression of myosin heavy-chain mRNA in muscles of mastication during functional advancement of the mandible in pigs. Arch Oral Biol.

[39] Gedrange T, Gredes T, Spassov A, Mai R, Kuhn DU, Dominiak M (2013). Histological changes and changes in the myosin mRNA content of the porcine masticatory muscles after masseter treatment with botulinum toxin A. Clin Oral Investig.

[40] Cao R, Huang F, Wang P, Chen C, Zhu G, Chen L (2015). Chronic sleep deprivation alters the myosin heavy chain isoforms in the masseter muscle in rats. Br J Oral Maxillofac Surg.

[41] Eriksson PO, Thornell LE (1983). Histochemical and morphological muscle-fibre characteristics of the human masseter, the medial pterygoid and the temporal muscles. Arch Oral Biol.

